# Prevalence and Associated Factor of Brown Adipose Tissue: Systematic Review and Meta-Analysis

**DOI:** 10.1155/2020/9106976

**Published:** 2020-06-16

**Authors:** Misganaw Gebrie Worku, Wullo Sisay Seretew, Dessie Abebaw Angaw, Getayeneh Antehunegn Tesema

**Affiliations:** ^1^Department of Human Anatomy, University of Gondar, College of Medicine and Health Science, School of Medicine, Gondar, Ethiopia; ^2^Department of Epidemiology and Biostatistics, University of Gondar, College of Medicine and Health Science, Institute of Public Health, Gondar, Ethiopia

## Abstract

**Background:**

Brown adipose tissue generates heat instead of storing energy. It is important in the regulation of body weight, and individual variation in adaptive thermogenesis can be attributed to variations in the amount or activity of BAT.

**Objective:**

The objective of this study was to systematically review different articles to assess the prevalence of BAT and its associated factors and relation with obesity and diabetes mellitus.

**Methods:**

A systematic review and meta-analysis were employed on published research works from different electronic databases using keywords. Cross-sectional studies and a few experimental studies were included for systematic review, and only studies done on human population were used for quantitative analysis. Twenty-two peer-reviewed papers were included in the systematic review, and eight papers were used for the meta-analysis for estimation of pooled prevalence of brown adipose tissue using selection criteria.

**Results:**

The pooled prevalence of brown adipose tissue among adults was 6.97% (95% CI: 6.51–7.43), and it was 7.4% (95% CI 6.51-7.43) after sequential omission of a single study. The heterogeneity in estimating the pooled prevalence among the studies was statistically significant (Cochran *Q* test, *P* < 0.001, *I*^2^ = 71.2%), and after sequential omission of a single study, it becomes Cochran *Q* test, *P* = 0.065, *I*^2^ = 49.4%. The brown adipose tissue activity was significantly lower in overweight or obese subjects than in lean subjects.

**Conclusion:**

The percentage of adult individuals with brown adipose tissue was high, and its activity was reduced in obese individuals. Although it is reduced in amount, still it presents in obese individuals. So, activation of the brown adipose tissue in adult and older individuals should be a target for the treatment of obesity.

## 1. Introduction

Positive energy balance is the main cause of obesity, diabetes, and associated metabolic disorder [1]. Excessive energy is stored as white adipose tissue in our body which contributes to obesity. In contrary, brown adipose tissue generates heat energy [1, 2]. The stimulation of brown adipose tissue increases the intake of glucose and consumption of energy, which may be targeted for treatment of obesity and metabolic disorder [2]. It is the site of adaptive thermogenesis that is responsible for regulating energy consumption and body fat [1]. The inverse relationship between body fatness and BAT activity suggests the protective role of brown adipose tissue against obesity [3]. In infants, there is a high prevalence of BAT, but adults have only less BAT localized in a specific region of the body. This decreased prevalence of BAT mass and activity in adults leads to the development of obesity and different metabolic disorders [1, 4]. Activation of BAT is associated with less prevalence of central obesity and hepatic fat [5], and its key function is to treat diabetes mellitus and obesity through energy consumption mechanisms [6]. Age-related decline in BAT activity induces excessive fat accumulation [7], which is also inversely associated with BMI [5]. The reversibility of brown adipose tissue recruitment and its mechanism of effect on energy metabolism were not researched in humans [8].

Large interindividual differences in cold-induced and diet-induced adaptive thermogenesis exist in animals and humans which have a large impact on long-term energy balance and body weight, which might lead to the susceptibility to obesity [9]. Individuals with autonomous BAT transplantation had a significant improvement in glucose tolerance test compared with controls [10], and it is involved in energy expenditure to control adiposity [7]. The sex of an individual and BMI had a strong association with BAT activity [11].

It is highly deposited in our body region from the anterior neck to the thorax region, and women have a greater mass of brown adipose tissue with a ratio of greater than 2 : 1 [5]. The prevalence of cold-activated glucose uptake in our body was higher in winter season and among young individuals [2]. The average standard uptake value (SUV) among BAT-positive individuals was 2- to 3-fold higher than that in BAT-negative individuals, and these BAT-negative individuals on PET/CT scan may show characteristic of BAT on histological examination [12].

Cold exposure results in an increase of BAT activity and concomitant decrease in body fat mass, indicating negative correlation between BAT activity and body fat accumulation [7]. Activation of BAT in obese and DM mice by b3-adrenergic receptor agonists results in weight loss and a decrease in blood glucose [6].

Activated BAT in the supraclavicular region was detected in 8.8% of patients, which is the area of high accumulation of brown adipose tissue [13]. In obese individuals, there is decreased cold-induced thermogenesis with less prevalence of brown adipose tissue, and in BAT-positive subjects, the mean energy consumption was increased considerably compared with that in BAT-negative individuals [14]. Another study reported that BAT activity was observed in 96% of subjects during cold exposure and the majority of obese individuals showed signs of BAT activity, but the mean BAT activity was slightly lower [15]. Despite physical exercise being considered as the most effective and healthy way of increasing energy consumption, many people did not perform the limited guidelines for sustaining an active lifestyle. So the search for a safe way to increase the metabolic rate for body weight maintenance is required [16], and BAT is currently a target for this and another metabolic disorder treatment via metabolization. Despite the potential contribution of BAT to metabolism, its mechanism still remains unclear due to the challenge of rate of BAT detection in humans [2]. Different studies reported that less than half of the study subjects had active BAT [5, 12, 13, 15, 17, 18], and this high prevalence of BAT was significantly correlated with age, sex, BMI, temperature, and seasonal variability. The prevalence of active BAT in females was higher than in males with a ratio of 1.74 : 1 and 2 : 1, respectively [17–19]. The prevalence of BAT among obese people was between 28% and 54%, while prevalence among lean individuals was found to be higher [18, 20].

Active BAT has been inversely correlated with age, outdoor temperature, beta-blocker usage, and BMI [5], and patients with positive BAT had lower body mass index and increased energy expenditure [10]. Also in winter, BAT prevalence increased compared to summer and inversely related to BMI and total and visceral fat areas [2].

In some studies, BMI and body fat percentage had negative associations with BAT prevalence, while resting metabolic rate had a strong positive correlation [15, 19, 21]. Individual variations in energy expenditure had significant effects on body weight, and several studies reported low energy expenditure leads to a gain in body weight [2, 9, 21], so BAT-induced thermogenesis could be a target for antiobesity therapies. Several studies had indicated BAT is occasionally present and active in adult humans [12, 19, 21], and other studies indicated BAT is related to the body mass index [3, 5, 11, 22]. So the review of different studies to assess and summarize the overall therapeutic effect of brown adipose tissue in the treatment of obesity and other metabolic disorder is very crucial.

## 2. Methods

### 2.1. Searching Strategy

**Figure 1:**
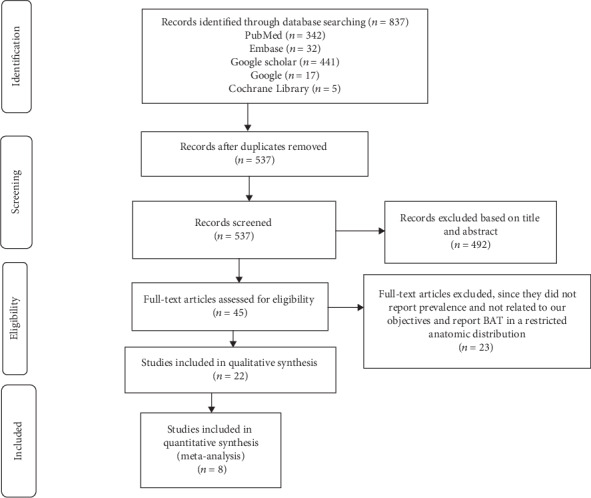
Flow diagram of studies included in the systematic review and meta-analysis.

We followed the methods of Li et al. [23] in writing the methodological part of this systematic review and meta-analysis. A comprehensive search was carried out to identify potentially relevant articles from different electronic databases. PubMed, direct Google, advanced Google Scholar, and Cochrane Library were used to identify articles. All published papers entitled in prevalence of BAT, associated factors, and its association with obesity and other metabolic disorder were included for the systematic review and meta-analysis. A study done at all age groups published across different parts of the world was used to select papers, but only studies at adult age groups were included for the quantitative analysis. The search language was limited to English, and we used a combination of keywords such as adipose tissue, brown adipose tissue, and adiposity for searching of different articles. The title and abstract were critically reviewed by two authors (MG) and (GA) after downloading the papers. The disagreement between the two reviewers was resolved by consensus, or the third reviewer (WS) decided against the inclusion of the article. The full document was thoroughly read and reread to include the paper for both qualitative and quantitative analysis (Figure 1).

### 2.2. Criteria for Inclusion and Exclusion

Studies included in our study were population-based study, a cross-sectional study, and experimental study with clear information on sample size and total prevalence of brown adipose tissue. A study published in the pediatric age group was included in the systematic review, but not in meta-analysis. We excluded studies that investigated specific populations, volunteers, and pediatric age groups (age < 18 years). Studies done at specific seasons and those intentionally exposed to different temperatures to induce BAT were excluded from quantitative analysis, but the effect was explained in the qualitative synthesis. For multiple studies on the same population, only the study that reported the most detailed data was included.

### 2.3. Data Extraction and Quality Assessment

All articles searched from electronic databases were combined in EndNote, and duplicates were removed. Two researchers (MG and GA) independently assessed the titles and abstracts and reviewed the full text of the eligible citations, and for any discrepancy, the third reviewer (DA) made the final decision. For each included study, two researchers (DA and WS) independently extracted the following information: general information (e.g., first author, title, journal, and publication year), study characteristics (including study period, study area, study design, sample source, sample selection method, and sample size), and all possible participant information. Two researchers (MG and GA) independently assessed the quality of each included study using NOS quality assessment tools. Only when the reviewers agreed was the study included in the qualitative and quantitative analysis. The retained articles were required to have a minimum quality score requirement.

### 2.4. Statistical Analysis

We used a systematic approach to estimate the pooled prevalence of brown adipose tissue from all eligible studies. A fixed-effect model was selected to summarize the prevalence of brown adipose tissue, using statistical tests for heterogeneity. Heterogeneity among studies was assessed using Cochran's *Q* test and *I*^2^ statistic. Since the data showed moderate heterogeneity (*I*^2^ < 75%), a fixed-effect model was used. Subgroup analyses by geographic region and the year of study were performed to address heterogeneity and to have pooled prevalence across different regions and estimation of prevalence of BAT at different periods. Additionally, sensitivity analysis (i.e., recalculating the pooled estimate by omitting studies) was performed to assess the influence of any particular study on the pooled prevalence. Publication bias was assessed using Egger's test. The significance level was set at a *P* value of less than 0.05. All statistical analyses were performed using Stata version 11.0 and Excel 2013.

## 3. Results

### 3.1. Search Results

A total of 837 articles were retrieved from different electronic databases. After removal of 300 duplicate articles, 492 of the studies were excluded based on review of the title and abstract. From these, 45 publications were considered for full-text review and 23 of the studies were excluded because they did not report the total prevalence clearly and some article estimates the prevalence of brown adipose tissue in a specific anatomical location of the body. Finally, 22 peer-reviewed papers were included for qualitative analysis. After eligibility evaluation, 8 papers which report the prevalence of brown adipose tissue in the whole body region, rather than a specific anatomical region, were retained for quantitative analysis, two in the UK, two in the USA, two in Australia, one in Germany, and one in Canada. The study selection process and flowchart of the literature search are shown in the diagram (Figure 1).

### 3.2. Prevalence

The prevalence of brown adipose tissue in different studies ranged between 5.8% and 17.6%. The information from selected papers with their prevalence was described and presented in Table 1.

**Figure 2:**
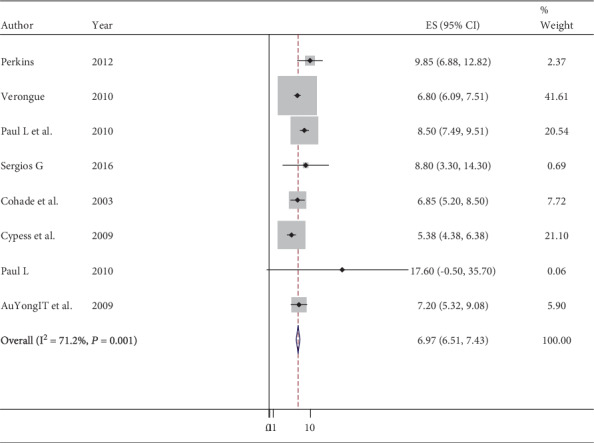
Forest plot indicates the prevalence of brown adipose tissue among adult individuals.

**Figure 3:**
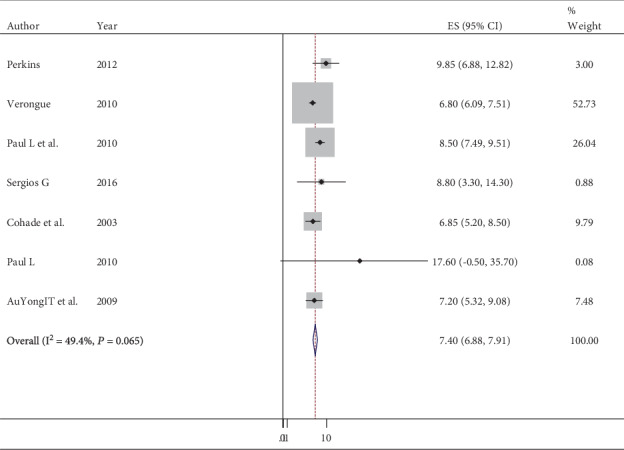
Forest plot indicates the prevalence of brown adipose tissue after the sequential omission.

A meta-analysis for the prevalence of eight peer-reviewed papers was done using Stata version 11, and the pooled prevalence of brown adipose tissue among adult patients was 6.97% (95% CI: 6.51–7.43) (Figure 2). Also, the heterogeneity in estimating the pooled prevalence among the studies was statistically significant; the Cochran *Q* test, *P* < 0.001, *I*^2^ = 71.2%. After sequential omission of a single study by sensitivity analysis, the prevalence of brown adipose tissue became 7.4% (95% CI: 6.88-7.91) and Cochran *Q* test, *P* = 0.065, *I*^2^ = 49.4% (Figure 3).

### 3.3. Subgroup Analysis

According to the subgroup analysis done by year of the study, slightly higher prevalence of active brown adipose tissue was observed in studies conducted after 2009 with the prevalence of 7.477% and 95% CI (6.911, 8.044), and the prevalence for the study done before 2009 was found to be 6.016% with 95% CI (5.24, 6.79). Subgroup analysis was also done by different geographical regions, and the highest prevalence of active brown adipose tissue was observed in the study conducted in Australian countries (8.528%, 95% CI (7.52, 9.536)). The pooled prevalence of brown adipose tissue in America and Europe was 6.38% with 95% CI (5.83, 6.925) and 8.02% with 95% CI (6.496, 9.552), respectively (Table 2).

### 3.4. Publication Bias

A review of the funnel plots did not rule out the potential for publication bias of brown adipose tissue, and it was assessed using Egger's test. The estimated bias coefficient was 1.26 (Egger bias *B* = 1.26: 95% CI: -1.6–4.1) with standard error of 1.16. Thus, the test provides no evidence for the presence of small-study effects (Table 3).

## 4. Discussion

The prevalence of brown adipose tissue ranged widely across studies. In the current systematic review and meta-analysis, the pooled estimate of prevalence of brown adipose tissue was 6.97% (95% CI: 6.51, 7.43), which is consistent with a study done in Canada (6.8%), USA (6.85%), and UK (7.2%) [5, 23]. The result of this study was higher than the prevalence reported in United States (5.38%) [10]. Several studies recorded high prevalence of active brown adipose tissue in Australia (17.6%), UK (9.85%), Germany (8.8%), and Australia (8.5%) (12, 13, 14).

The detection rate of activated brown adipose tissue is related to outdoor temperature, age, sex, and seasonal variability. Mostly, high prevalence of active brown adipose tissue is found in the first decades of life and after prolonged exposure to cold which is higher than the prevalence of brown adipose tissue measured at the thermo neutral conditions. The pooled estimate of the prevalence of brown adipose tissue in European countries was higher than that in Australia and America, suggesting that the prevalence of brown adipose tissue varies across different regions. This variation may be associated with seasonal variation and PET-CT scan type and sensitivity used to detect brown adipose tissue. At normal temperature, the rate of brown adipose tissue activity may be similar both in BAT-negative and BAT-positive individuals. But after cold exposure, the rate of activity may be higher among brown adipose tissue-positive individuals [19]. This variation may be due to the difference in the outdoor temperature and less sensitivity of PET/CT scan. The rate of detection of activated brown adipose tissue in females was higher than that in males [5], which is consistent with a study conducted in the United Kingdom (9.85%), with higher prevalence in females than males. The prevalence of brown adipose tissue was higher in the pediatric age group (12.73%) relative to adults. This indicates that at the pediatric age group the prevalence of brown adipose tissue is higher in males than in females, in contrast to the adult and higher age groups [17]. Obese individuals had less prevalence of BAT than lean individuals indicating the thermogenic effect of brown adipose tissue for body weight reduction. The higher prevalence of BAT and cold-activated FDG uptake was observed in the supraclavicular regions, suggesting the higher prevalence of brown adipose tissue in the specific anatomical region and young people. There was an important correlation between the BAT detection rate and the seasonal variability. The rate of detection of brown adipose tissue also differs with seasonal variability, which was higher in winter and lower in summer, which is about three times lower. This seasonal variability in the detection rate of activated brown adipose tissue may be due to the difference in temperature [2]. In the studies included in our analysis, there was a higher prevalence of metabolically active brown adipose tissue identified by PET-CT 18F FDG uptake and it was higher in females than in males in nearly half of the sample. There is no simple explanation for this higher prevalence of metabolically active brown adipose tissue in women than in men, and a limited number of morbidly obese patients had been detected with active BAT in some study, suggesting that morbid obesity is associated with low BAT activity. Also, the data showed that BAT was present in some subjects even in these groups and can be activated by cold exposure indicating body composition is highly linked to BAT activity [15, 21].

## 5. Conclusion

In this systematic review and meta-analysis, we can conclude that the percentage of an adult with brown adipose tissue was high, but in humans who are overweight or obese, its prevalence is found to be small. Brown adipose tissue is metabolically important in humans and still present in overweight or obese subjects with limited amount and makes it a goal for obesity treatment. Different studies in this systemic review have showed that adults had functional BAT, with thermogenesis and energy-reducing capacity. Human BAT response to cold stimulation is regulated by adrenergic stimulation. Combining this knowledge with recent advances in understanding the differentiation between BAT has created new interest in this tissue as a possible therapeutic approach to metabolic diseases. While many questions remain unanswered regarding the practicality and durability of such treatments, we encourage that therapies targeting BAT thermogenesis should be available as therapies for obesity in the near future.

## Figures and Tables

**Table 1:** Characteristics of included studies and prevalence of brown adipose tissue.

ID	Author	Year	Country	Population	Sample size	Sampling technique	Study design	Method of detection	Prevalence
1	Perkins	2012	UK	Hospital based	386	Consecutive	Cross-sectional	18F-FDG PET-CT scan	9.85%
2	Verongue	2010	Canada	Hospital based	4842	Consecutive	Cross-sectional	18F-FDG PET-CT scan	6.8%
3	PaulL et al.	2010	Australia	Hospital based	2934	Consecutive	Cross-sectional	18F-FDG PET-CT scan	8.5%
4	Sergios G	2016	Germany	Hospital based	102	Consecutive	Cross-sectional	18F-FDG PET-CT scan	8.8%
5	Cohade et al.	2003	USA	Hospital based	905	Consecutive	Cross-sectional	18F-FDG PET-CT scan	6.85%
6	Cypess et al.	2009	USA	Hospital based	1972	Consecutive	Cross-sectional	18F-FDG PET-CT scan	5.38%
7	Paul L	2010	Australia	Hospital based	17	Consecutive	Cross-sectional	18F-FDG PET-CT scan	17.6%
8	AuYong IT et al.	2009	UK	Hospital based	724	Consecutive	Cross-sectional	18F-FDG PET-CT scan	7.2%

**Table 2:** Subgroup analysis for the prevalence of BAT done by geographical regions.

Study	ES	95% conf. interval		% weight
*Europe*				
Perkins	9.8500	6.877, 12.823		2.37
Sergio G	8.800	3.302, 14.298		0.69
AuYong et al.	7.200	5.317, 9.083		5.90
Subtotal I-v pooled ES	8.024	6.496, 9.552		8.96
*America*				
Verongue	6.800	6.091, 7.509		41.61
Cohade et al.	6.850	5.204, 8.496		7.72
Cypess et al.	5.380	4.384, 6.376		21.10
Subtotal I-v pooled ES	6.380	5.835, 6.925		70.43
*Australia*				
PaulL et al.	8.500	7.491, 9.509		20.54
Paul L	17.600	-0.503, 35.703		0.06
Subtotal I-v pooled ES	8.528	7.521, 9.536		20.61
Overall I-v pooled ES	6.970	6.513, 7.427		100.00
Test(s) of heterogeneity	Heterogeneity statistics	Degree of freedom	*P* value	*I* ^2∗∗^
Europe	2.26	2	0.323	11.6%
America	5.53	2	0.063	63.9%
Australia	0.97	1	0.325	0.0%
Overall	24.28	7	0.001	71.2%
Overall test for heterogeneity between subgroups				
	15.51	2	0.000	

*I* ^2∗∗^: the variation in ES attributable to heterogeneity.

**Table 3:** Egger's test for the assessment of publication bias.

Egger's test	95% conf. interval				
Std-Eff	coef	std. err	*P* value	Lower	Upper
Slope	6.28	0.764	0.000	4.4122	8.153
Bias	1.26	1.1583	0.319	-1.5766	4.091

## Abbreviations

BAT: Brown adipose tissue
SD: Standard deviation
UOG: University of Gondar
PET: Positron emission tomography
CT: Computed tomography
BMI: Body mass index
FDG: Fludeoxyglucose.

## Supplementary Materials

Supplementary materials The supplementary file contains “The PRISMA 2009 Checklist.”
