# Supplementary material for: Prevalence and Associated Factor of Brown Adipose Tissue: Systematic Review and Meta-Analysis
Source: Biomed Res Int. 2020 Jun 16;2020:9106976. doi: 10.1155/2020/9106976 (PMC7317326; doi:10.1155/2020/9106976)
Supplement: Supplementary materials — The supplementary file contains “The PRISMA 2009 Checklist.” [file 9106976.f1.doc]

| **Section/topic** | **#** | **Checklist item** | **Reported on page #** |
| --- | --- | --- | --- |
| **TITLE** | | |  |
| Title | 1 | Prevalence and associated Factor of brown adipose tissue: systematic review and meta-analysis. | 1 |
| **ABSTRACT** | | |  |
| Structured summary | 2 | ABSTRACT  Background: The objective of this study was systematic review of different articles to assess the prevalence of BAT; associated factor and its relation with obesity diabetes mellitus.  Methods: A systematic review was employed on published research works from different electronic databases using key words. Across-sectional studies and a few experimental studies were included for systematic review and only studies done on human population were used for quantitative analysis. Twenty-two peer-reviewed papers were included in systematic review and eight papers were used for meta-analysis for estimation of pooled prevalence using selection criteria.  Results: The pooled estimation of prevalence of BAT among adults were 6.97 %( 95% C.I: 6.51–7.43) and it was 7.4 %( 95% CI 6.51-7.43) after sequential omission of a single study. The heterogeneity in estimating the pooled prevalence among the studies was statistically significant; Cochran Q test, P < 0.001, I2=71.2% and Cochran Q test, P=0.065, I2=49.4% after sequential omission of a single study. The activity was significantly lower in the overweight or obese subjects than in lean subjects.  Discussion/Conclusions: The percentage of adult individuals with brown adipose tissue was high and its activity was reduced in obese. Although it is reduced yet present in obese individuals, make it a target for treatment of obesity. | 1 |
| **INTRODUCTION** | | |  |
| Rationale | 3 | Brown adipose tissue is metabolically important in human and the fact that it is reduced yet present in most overweight or obese subjects make it a target for the treatment of obesity. Different studies had reported adult humans have functional BAT, a tissue that has a tremendous capacity for obesity-reducing thermogenesis. The prevalence of brown adipose tissue varies among different studies included in this study, so this study try to estimate the pooled prevalence of brown adipose tissue among included studies and factors related to it. |  |
| Objectives | 4 | The objective of this study was systematic review of different articles to assess the prevalence of BAT; associated factor and its relation with obesity diabetes mellitus. |  |
| **METHODS** | | |  |
| Protocol and registration | 5 | It is registered in Prospero registration for international systematic review and meta-analysis with registration number of 157460. |  |
| Eligibility criteria | 6 | A study to be included in our study it should be Population-based study, a cross-sectional study, experimental and study that provide sufficient information of sample size and total prevalence of brown adipose tissue. To be included in the quantitative analysis the study must be population based and age of the study participant should be greater than or equal to eighteen. All articles which used institutional based cross sectional study design and which clearly report the prevalence and associated factors were included. We excluded studies that investigated specific population, volunteers and pediatric age group. For multiple study on the same population, only the study that reported the most detailed data was included. | 4 |
| Information sources | 7 | The source of information for this systematic review and meta-analysis from different electronic data base including, PubMed, direct google, google scholar and chochrean library. The information for this study searched fromoctober29/10/2019 up to december21/12/2019. |  |
| Search | 8 | First all available articles are searched using key words and mesh terms if available, then this searched articles are screened using different quality assessment tools starting from the topic. And finally the study that clearly reported the prevalence and that directly related to our objective are included ti the systematic review. | 4 |
| Study selection | 9 | A total of 837 articles were retrieved from different electronic search engines. In a primary screening process 300 were excluded because of duplication and 492 of the publications were excluded after reading the title and abstract. The retained 45 publications were screened after obtaining and reading the full PDF text and 23 of the paper were excluded because they didn’t report the total prevalence clearly and also some article estimate the prevalence of brown adipose tissue in restricted anatomical position. Finally 22 peer-reviewed papers were included for qualitative analysis and after eligibility evaluation, finally, 8 papers were retained for quantitative analysis. | 4-5 |
| Data collection process | 10 | The data extracted from different electronic database using electronic search engine only and data from reports are not available and didn’t included in this systematic review and meta-analysis. And all the article and the data are freely available from this electronic database | 4-5 |
| Data items | 11 | This systematic review and meta-analysis aimed in assessing Prevalence of brown adipose tissue and its relation with the variable, age, sex, height, weight, body mass index, seasonal variation. |  |
| Risk of bias in individual studies | 12 | For each included study two researcher independently asses the quality of the study by considering design of the study, sample source, sample selection method and the sample size. Finally the quality of each study assessed with JBI quality assessment tools. | 5 |
| Summary measures | 13 | Pooled prevalence of brown adipose tissue. | 6 |
| Synthesis of results | 14 | We used a systematic approach to calculate the pooled estimate prevalence of brown adipose tissue from all eligible studies. A fixed effect model was selected to summarize the prevalence of brown adipose tissue, using statistical tests for heterogeneity. Heterogeneity among studies was assessed using Cochran’s Q test and I^2^ statistic, which shows the percentage of variation across studies. Since the data showed low or moderate heterogeneity (I^2^ < 75 %), a fixed-effect model was used. | 6 |

Page 1 of 2

| **Section/topic** | **#** | **Checklist item** | **Reported on page #** |
| --- | --- | --- | --- |
| Risk of bias across studies | 15 | In this study, publication bias was also assessed using Egger test. Sensitivity analysis was performed by sequential omission of individual studies. The pooled prevalence from sequential omission was significantly changed. | 8 |
| Additional analyses | 16 | Sub-group analysis were done by year of study and also done by different continent.The sub group analysis showed the variation of pooled prevalence across different geographical region and periods. Additionally, sensitivity analysis (i.e., recalculating the pooled estimate by omitting studies) was performed to assess the influence of any particular study on the pooled estimate. | 8 |
| **RESULTS** | | |  |
| Study selection | 17 | A total of 837 articles were retrieved from different electronic search engines. In a primary screening process 300 were excluded because of duplication and 492 of the publications were excluded after reading the title and abstract. The retained 45 publications were screened after obtaining and reading the full PDF text and 23 of the paper were excluded because they didn’t report the total prevalence clearly and also some article estimate the prevalence of brown adipose tissue in restricted anatomical position. Finally 22 peer-reviewed papers were included for qualitative analysis and after eligibility evaluation, finally, 8 papers were retained for quantitative analysis two in UK, two in USA, two in Australia one in German and one in Canada. | 6 |
| Study characteristics | 18 | For each study included the prevalence of brown adipose tissue, sample size, sampling technique, year of study, country, and factors that affect the prevalence of brown adipose tissue are recorded. | 6 |
| Risk of bias within studies | 19 | For each included study two researcher independently asses the quality of the study by considering design of the study, sample source, sample selection method and the sample size. Finally the quality of each study assessed with JBI quality assessment tools. |  |
| Synthesis of results | 20 | Eight Peer-reviewed papers were used on the analysis and interpretation of data. The information from selected papers was described with their prevalence and significantly associated factor described and presented on table (1) constructed in Microsoft word. A meta-analysis for the prevalence of eight peer-reviewed paper was done using Stata version 11 and the pooled estimation of the prevalence of active brown adipose tissue among adult’s patients was 6.97 %( 95% C.I: 6.51–7.43). Also, the heterogeneity in estimating the pooled prevalence among the studies was statistically significant; Cochran Q test, P < 0.001, I^2^=71.2%. After sequential omission of a single study by sensitivity analysis the prevalence of brown adipose tissue became 7.4% (95%CI: 6.51-7.43) and Cochran Q test, P=0.065, I^2^=49.4%. | 6 |
| Risk of bias across studies | 21 | In this study, publication bias was also assessed using Egger test. Sensitivity analysis was performed by sequential omission of individual studies. The pooled prevalence from sequential omission was significantly changed. | 6 |
| Additional analysis | 22 | According to the sub-group analysis done by year of study slightly higher prevalence of active brown adipose tissue was observed in studies that published after 2009 with prevalence of 7.477% and 95%CI(6.911, 8.044) and the prevalence for the study done before 2009 was found to be 6.016% with 95%CI(5.24, 6.79). Subgroup analysis was also done by different continent and the highest prevalence of active brown adipose tissue was observed in the study conducted in Australia countries (8.528%, 95%CI (7.52, 9.536)). The pooled prevalence of brown adipose tissue in America and European was 6.38% with 95%CI (5.83, 6.925) and 8.02% with 95%CI (6.496, 9.552) respectively. | 7 |
| **DISCUSSION** | | |  |
| Summary of evidence | 23 | The prevalence of Brown adipose tissue varied widely across studies. In the current systematic review and meta-analysis the pooled estimation of the prevalence of active brown adipose tissue of adult patients was 6.97 %( 95% C.I: 6.51–7.43). Detection of activated brown adipose tissue significantly associated with outdoor temperature, age, sex, and seasonal variation. Mostly high prevalence of active brown adipose tissue is detected in the first decades of life and after extended cold exposer which is higher than prevalence of brown adipose tissue measured at the Thermo neutral conditions. The pooled estimate of prevalence of brown adipose tissue in European countries was higher as compared to those of Australia and America, which indicates that there is variation in the prevalence of brown adipose across different continents. This variation may be associated with seasonal variation and the type and sensitivity of PET-CT scan used for the detection of active brown adipose tissue. | 9 |
| Limitations | 24 | As a limitation, some of the studies didn’t quantify the prevalence of brown adipose tissue and also the less sensitivity of the PET-CT used to assess 18F FDG uptake which may be negative in some of the studies which are positive after histological examination. Also the rate of detection of brown adipose is affected by seasonal variation, it is difficult to estimate the true prevalence of brown adipose tissue. Therefore it is recommended to have an appropriate and standard method for the detection of activity of brown adipose tissue. |  |
| Conclusions | 25 | From this systematic review, we can conclude that the Percentage of adult with brown adipose tissue was high, but its prevalence and activity is reduced in human who is overweight or obese. Brown adipose tissue is metabolically Important in human and the fact that it is reduced yet present in most overweight or obese subjects make it a target for the treatment of obesity. Different studies in this systemic review had conclusively showed adult humans have functional BAT, a tissue that has a tremendous capacity for obesity-reducing thermogenesis. It is now clear that human BAT responds to cold stimulation under both acute and chronic conditions, is regulated by adrenergic stimulation, and can be monitored using noninvasive imaging via PET/CT. Combining this knowledge with recent advances in understanding BAT differentiation has created new interest in this tissue as a possible therapeutic approach for metabolic diseases. Although many questions remain unanswered regarding practicality, and durability of such treatments, we encouraged that classical therapies targeting BAT thermogenesis should be available in the near future as therapies for obesity. | 9 |
| **FUNDING** | | |  |
| Funding | 26 | No funding body involved in systematic review and meta-analysis of this study. |  |

*From:*  Moher D, Liberati A, Tetzlaff J, Altman DG, The PRISMA Group (2009). Preferred Reporting Items for Systematic Reviews and Meta-Analyses: The PRISMA Statement. PLoS Med 6(7): e1000097. doi:10.1371/journal.pmed1000097

For more information, visit: **www.prisma-statement.org**.

Page 2 of 2
